# Supplementum Plantarum Systematis Vegetabilium editionis decimæ tertiæ, Generum Plantarum editionis sextæ, et Specierum Plantarum editionis secundæ

**Published:** 1784

**Authors:** 


IV. *Supplementum Plantarum Syſtematis Vegetabilium editionis decimæ tertiæ, Generum Plantarum editionis ſextæ, et Specierum Plantarum editionis ſecundæ, editum a* Carolo a Linné, *Med. Doct. Med. et Botan. Prof. Upſal. Hort. Botan. Præf.* 8vo. Brunſvigæ, 1781. p. 467.

IN a well-written preface to this work the editor informs us, that it was begun by his father, who deſcribed and arranged the plants he received a little before his death from Surinam and Africa. To theſe the editor has been enabled enabled to make confiderable additions by the kindnefs of his botanical friends.

In claffification our author has made one change, by excluding the clafs *Polygamia.* His reafons for this we fhall give in his own words. "Progreffu temporis experientia do-"cuit illam claffem potius damnum quam uti-"litatem attuliffe methodo. Laceratio Gene-"rum Naturalium inde orta, variatio, quam "cultura interdum in fexu efficit, et in primis "difficultates, quando ex his hermaphroditus "five abortiens flos fe unice offert, fatis fu-"perque neceffitatem hujus mutationis mihi "probarunt. Vix tamen aufus fuiffem pro-"priæ confidere experientiæ, atque illa unice "nifus hanc inftituere reformationem, nifi il-"lud ipfum, quod attuli, a celeberrimis Bo-"tanicis, (in primis qui fequiore ætate pere-"grinati funt) fuerit obfervatum, quorum con-"filio uti in hoc puncto mihi gratum fuit. "Omnia itaque nova genera in reliquis collo-"cavi claffibus, fed antiquorum generum fpe-"cies fuis collocatas locis volui ad evitandam "majorem confufionem."

He profeffes to have been fparing in forming new genera. "Genera annifus fum," fays he, "quantum fieri potuit, pauca formare nova;

" sed quæ herbæ potuerunt transferri ad ali-

" quod genus jam antea cognitum sine damno

" characteris essentialis, illuc quoque easdem

" assignavi, quantumvis singularem nactus es-

" sem characterem in iisdem dignoscendis:"

and a little farther on he adds, " Satis su-

" perque expertus sum, quod multa genera

" inutiliter scientiam extendant, affines sepa-

" rent, multiplicent difficultates in iisdem in-

" ternoscendis, et impedire plerumque soleant

" perspicuitatem in charactere essentiali, quo

" evenit, ut genera sensim per nova inventa

" ingrediantur se invicem, nec facile discerni

" possint. Debet Botanicus in novo genere

" formando ante omnia exquirere ejus affinita-

" tem, ac studiose, quantum fieri potest, sarta

" tectaque habere genera naturalia, seque hoc

" in casu subjicere Naturæ legibus."

The preface is followed by a description of ninety-three genera, which are either new, or such as have been imperfectly described before; after which the editor proceeds to the different species. Among these, the number of which amounts to 1403, we observe the *Myroxylon Peruiferum*, the tree which furnishes the balsam of Peru; — *Quassia Simaruba*, the bark of the root of which is the Simaruba bark of the shops;

ſhops ; — *Pſychotria Emetica*, the root of which our author ſuppoſes to be the true Ipecacuanha; — *Ignatia Amara*, the plant which bears the *Faba Sancti Ignatii* ; — *Myriſtica Officinalis* (nutmeg tree) ; — *Pterocarpus Santalinus*, the tree which yields the true red ſanders (*Santalum rubrum)*, and alſo a juice reſembling dragon's blood ; — *Hypericum bacciferum*, from the juice of which is prepared the *Gummi Gutta Americana* ; — *Jatropha Elaſtica*, the tree which yields elaſtic gum ; — *Mimoſa Catechu*, the plant from which Terra Japonica is extracted, (See *Med. Obſ.* & *Inq.* Vol. V.) ; — *Dioſpyros Ebenum*, the tree which furniſhes black ebony. — Several plants, of which deſcriptions have been publiſhed by eminent botanic writers, are intentionally omitted by the editor, as he profeſſes to deſcribe only ſuch as he himſelf has ſeen.

We obſerve that the names of many plants are changed ; and that ſome plants, formerly ſuppoſed to be different, and arranged under different genera or ſpecies, are now united into one ſpecies. Thus the *Caſſine capenſis, ramulis tetragonis, foliis petiolatis ovato oblongis, retuſis crenatis*, is the ſame plant as the *Caſſine Capenſis* and *Caſſine Barbara* of the *Syſtem. Veg.* ed. 13,

 p. 243 ; p. 243. A few ſpecies are divided into ſeveral. Thus a variety of *Othonna bulboſa* (Spec. Plant. ed. 2, p. 1309) is made a new ſpecies, and called *Othonna pinnata, foliis pinnatifidis; pinnis lanceolatis integerrimis decurrentibus.*

The peruſal of this work has excited in us freſh regret for the author's death, as we learn from it that he had formed a deſign of publiſhing a new edition of his father's *Genera Plantarum* and *Syſtema Vegetabilium*; an undertaking for which he ſeems to have been ably qualified.

